# Alzheimer’s Disease CSF Biomarkers as Possible Indicators of Tap-Test Response in Idiopathic Normal Pressure Hydrocephalus

**DOI:** 10.3390/brainsci13111593

**Published:** 2023-11-15

**Authors:** Efstratios-Stylianos Pyrgelis, George P. Paraskevas, Vasilios C. Constantinides, Fotini Boufidou, Myrto Papaioannou, Leonidas Stefanis, Elisabeth Kapaki

**Affiliations:** 1 1st Department of Neurology, School of Medicine, National and Kapodistrian University of Athens, Eginition Hospital, Vass. Sophias Ave. 74, 11528 Athens, Greece; stratospyrg@yahoo.gr (E.-S.P.); vassilis.kon@hotmail.com (V.C.C.); lstefanis@med.uoa.gr (L.S.); 2 1st Department of Neurology, Neurochemistry and Biological Markers Unit, School of Medicine, National and Kapodistrian University of Athens, Eginition Hospital, Vass. Sophias Ave. 74, 11528 Athens, Greece; geoprskvs44@gmail.com (G.P.P.); fboufidou@med.uoa.gr (F.B.); myrtop@yahoo.com (M.P.); 3 2nd Department of Neurology, School of Medicine, National and Kapodistrian University of Athens, “Attikon” University General Hospital, Rimini 1, 12462 Athens, Greece

**Keywords:** CSF biomarkers, total Tau, phospho-Tau, Aβ42, Aβ42/Aβ40 ratio, AD, Tap-test, idiopathic normal-pressure hydrocephalus

## Abstract

The aim of the present study is the evaluation of established Alzheimer’s disease (AD) cerebrospinal fluid (CSF) biomarkers in patients with idiopathic normal-pressure hydrocephalus (iNPH), both individually and as a total profile, and the investigation of their use as potential predictors of Tap-test responsiveness. Fifty-three patients with iNPH participated in the study. Aβ42, Aβ40, total Tau and phospho-Tau proteins were measured in duplicate with double-sandwich ELISA assays. Clinical evaluation involved a 10 m timed walk test before an evacuative lumbar puncture (LP) and every 24 h for three consecutive days afterwards. Neuropsychological assessment involved a mini-mental state examination, frontal assessment battery, 5-word test and CLOX drawing test 1 and 2, which were also performed before and 48 h after LP. Response in the Tap-test was defined as a 20% improvement in gait and/or a 10% improvement in neuropsychological tests. The Aβ42/Aβ40 ratio was found to be significantly higher in Tap-test responders than non-responders. Total Tau and phospho-Tau CSF levels also differed significantly between these two groups, with Tap-test responders presenting with lower levels compared to non-responders. Regarding the AD CSF biomarker profile (decreased amyloid and increased Tau proteins levels), patients with a non-AD profile were more likely to have a positive response in the Tap-test than patients with an AD profile.

## 1. Introduction

Hydrocephalus as a term comes from the conjunction of two Greek words “hydro” and “cephalus” which mean water and head, respectively, defining the accumulation of cerebrospinal fluid (CSF) in the ventricular system and subarachnoid spaces of the brain [1,2]. There are two types of hydrocephalus: non-communicating or obstructive, and communicating [1,2,3].

Normal-pressure hydrocephalus (NPH) is a type of communicating hydrocephalus, characterized by dilation of brain ventricles and normal CSF pressure more frequently affecting elderly patients, and is distinguished into secondary NPH and idiopathic NPH (iNPH) that has no apparent cause [2,4,5]. Clinically, iNPH is defined as a triad of symptoms, namely gait disorder, cognitive impairment, and urinary incontinence, as has been described by Adams and Hakim [6,7]. The main imaging characteristics of iNPH are ventriculomegaly (e.g., Evans index > 0.3) and lack of obstruction regarding CSF flow. Other imaging features include dilation of temporal horns, periventricular white matter lesions, narrow callosal angle, high tight convexity, focally enlarged sulci and dilation of Sylvian fissures [8]. There are two separate sets of diagnostic guidelines; one Japanese and one International. In both guidelines, iNPH diagnosis is based on the existence of both clinical and neuroimaging characteristics, with normal CSF opening pressure and a relatively slow progression of symptoms [9,10,11]. Its incidence ranges between 1.8 and 7.3/100,000 [12] and its prevalence ranges from 2.9% to 3.5% in patients with an age greater than 65 years [13,14].

Various pathogenetic theories have been suggested, but the exact pathophysiological mechanism remains unclear. The majority of theories converge on a vicious circle of abnormal resorption of the CSF leading to an impaired CSF micro-circulation and enlargement of the ventricles [5]. There is a scale that aims to evaluate and quantify the clinical status of iNPH patients: the so-called iNPH grading scale. It involves three distinct subfields which refer to gait impairment, cognitive impairment and urinary symptoms. Each subfield is rated between 0 and 4 depending on the extent of the disturbance, and subsequently, total scores range from 0 to 12, with higher scores relating to greater disturbances [15].

Its differential diagnosis includes a lot of entities that share some clinical and imaging features with iNPH, including other types of hydrocephalus, neurodegenerative dementias and vascular dementias. Due to the fact that iNPH usually affects the elderly, it is more often found to coexist with other conditions causing dementia, rather than existing as a pure syndrome [16].

Due to the fact that it is a potentially reversible condition, when appropriately selected patients are promptly treated with ventriculoperitoneal (VP) shunt or third ventriculostomy, its early diagnosis is of paramount importance [4,17,18]. One of the most well-established diagnostic tests and at the same time a prognostic factor for successful response to shunt surgery is the “Tap-test”, that includes a lumbar puncture (LP), removing 30–50 mL CSF, and the evaluation of possible clinical improvement afterwards. Gait disturbance seems to be the symptom that better responds to CSF drainage. It is a test with a high positive prognostic value, meaning that patients with a positive Tap-test response have a high probability of a good outcome after treatment with VP placement [5,18].

Consequently, proper selection of iNPH patients that may be improved when surgically treated is of paramount importance. Alzheimer’s disease (AD) is not only included in the differential diagnosis of iNPH [5,19], but also often coexists according to pathological studies [20,21]. Therefore, in an era when CSF biomarkers for the in vivo diagnosis of AD (decreased amyloid and increased Tau proteins levels) are well established, it would be interesting if they could also prove useful in aiding the selection of proper iNPH candidates for surgical intervention.

The purpose of the present study was the analysis of established AD CSF biomarkers in patients with iNPH; the comparison of their values, both individually and as a complete profile, between Tap-test responders and non-responders; and subsequently, their evaluation as a possibly helpful tool in iNPH prognosis.

## 2. Materials and Methods

### 2.1. Study Population

A total of 53 subjects were included in the study. All subjects were recruited prospectively during the years 2019–2021 among patients who presented to the 1st Department of Neurology of the National and Kapodistrian University of Athens at Eginition Hospital.

In order to be included in the study, patients had to fulfill the criteria of probable or possible iNPH according to the recent Guidelines for Management of Idiopathic Normal-Pressure Hydrocephalus [22,23]. Patients with a medical history or clinical or laboratory findings of other neurological or systemic disease with potential nervous system impairment were excluded. Patients with a history of traumatic brain injury were also excluded. All patients that were included in the study underwent brain MRI examination, and the neuroimaging data and their correlation with Tap-test response have been published elsewhere [24].

### 2.2. Ethical Issues

The study was in accordance with the ethical guidelines of the Declaration of Helsinki and had the approval of the local Ethical and Deontology committee of our hospital. All subjects and/or relatives gave informed consent for participation in the study.

### 2.3. Tap-Test Evaluation

An evacuative lumbar puncture was performed once in all patients of the study after their initial clinical and neuropsychological evaluation. Clinical evaluation included gait assessment via the 10 m timed walk test (a measurement of the time and steps required to walk a distance of ten meters), which was performed before and every 24 h after LP for three consecutive days [25].

Neuropsychological evaluation was performed before and 48 h after LP using (1) The Mini-Mental State Examination (MMSE) for global cognitive assessment [26]; (2) the Frontal Assessment Battery (FAB) for executive functions [27]; (3) the 5-word immediate and delayed recall (5WT) for memory [28]; and (4) the 15-point spontaneous and copy CLOX drawing (CLOX1 and 2, respectively) for executive functions (CLOX1) and constructional impairment (CLOX2) [29]. Clinical and cognitive evaluations were performed by the same neurologist experienced in the specific field.

Response to the Tap-test was outlined based on the following criteria: (1) a ≥20% improvement in gait tests and and/or (2) a ≥10% improvement in at least MMSE and FAB. In this way, patients were separated into two groups: Tap-test responders and non-responders [11,25].

### 2.4. CSF Sampling and Biomarkers’ Analysis

All patients underwent lumbar puncture in the morning, after overnight fasting, following established procedures based on recommendations for the standardization of pre-analytical confounding factors [30]. In brief, the CSF samples were afterwards collected in polypropylene tubes, centrifuged at 2000 g for 10 min at room temperature. They were immediately split into aliquots of 0.5 mL and then deep-frozen at −80 °C. Finally, every aliquot was thawed once right before its analysis. The opening pressure of CSF was measured, and patients with opening pressure higher than 20 cm H_2_O were excluded from the study. CSF biomarkers beta-Amyloid 1–42 (Aβ42), beta-Amyloid 1–40 (Aβ40), total Tau, and phospho-Tau (in threonine 181) were measured in duplicate with the use of commercially available ELISA kits from EUROIMMUN, applied in the fully automated analyzer EUROIMMUN Analyzer I (EUROIMMUN, Medizinische Labordiagnostika AG, Lübeck, Germany). Aβ42/Aβ40 and phospho-Tau/Aβ42 ratios were also calculated.

### 2.5. Statistical Analysis

All numerical data were tested for normality and homogeneity of variances using the Shapiro–Wilk and Brown–Forsyth tests, respectively. For the variables that did not have normal distributions and homogenous variances, we used nonparametric tests for statistical analysis. The Mann–Whitney U Test was used to investigate differences in the mean values of Aβ42, Aβ40, total Tau, phospho-Tau, Aβ42/Aβ40, and phospho-Tau/Aβ42 ratios among Tap-test responders and non-responders.

Categorical data were compared between groups using the χ^2^-test. All tests were performed using IBM SPSS Statistics^®^ version 23.0.0.0 (SPSS Inc., Chicago, IL, USA, 2013). All graphs were designed using GraphPad Prism^®^, version 8.43 (GraphPad Software Inc., La Jolla, CA, USA, 2020).

## 3. Results

The group of 53 patients that participated in this study was divided into two sub-groups, Tap-test responders and non-responders, depending on their performance in the Tap-test based on the criteria described above. Out of 27 Tap-test responders, 17 presented gait improvement, 4 had cognitive improvement and 6 had both.

Demographic and clinical characteristics regarding patients’ status before LP of each subgroup are shown in Table 1.

Clinical and neuropsychological data of responders and non-responders before and after LP are shown in Table 2 and Table 3, respectively.

**Figure 1:**
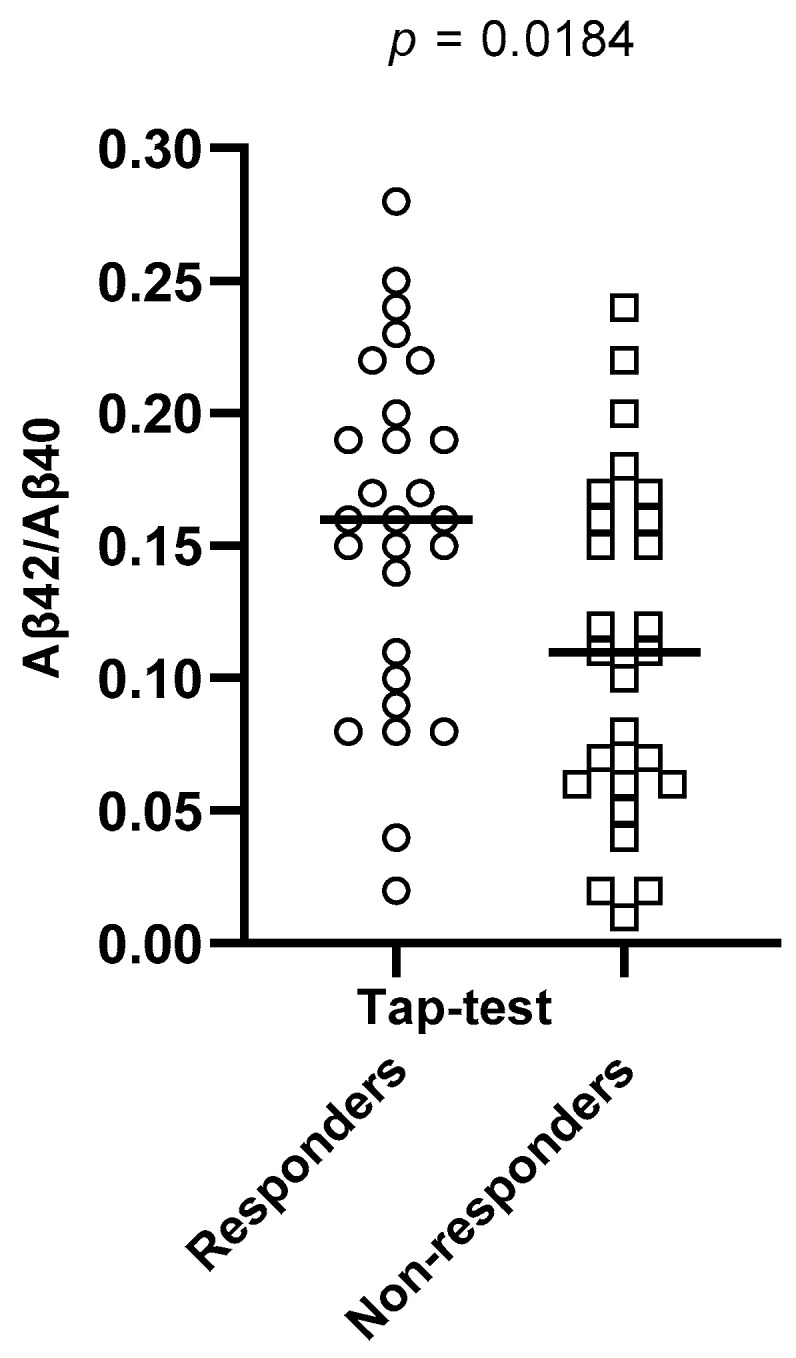
Aβ42/Aβ40 ratios differed significantly between Tap-test responders (depicted in circles) and non-responders (depicted in squares) (*p* = 0.0184). The median values and the range of these ratios’ values in the two groups are presented in this graph.

**Figure 2:**
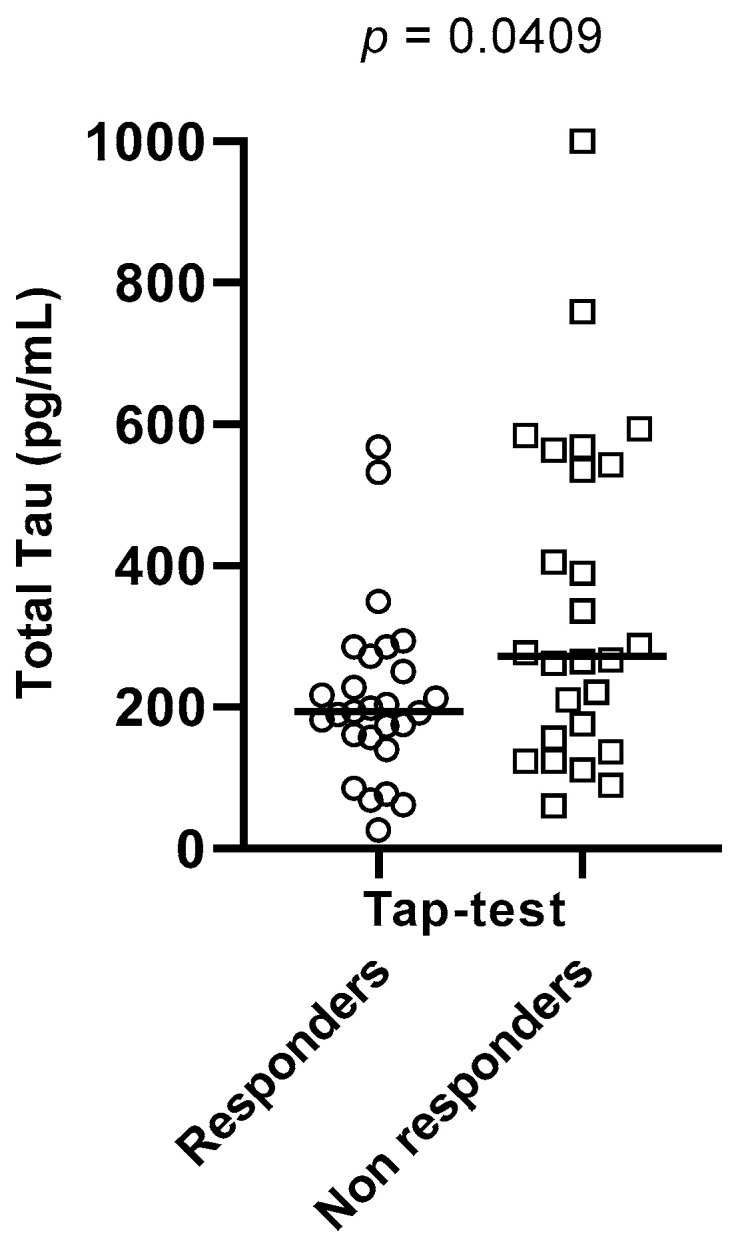
Total Tau levels differed significantly between Tap-test responders (depicted in circles) and non-responders (depicted in squares) (*p* = 0.0409). The median values and the range of total Tau values in the two groups are presented in this graph.

**Figure 3:**
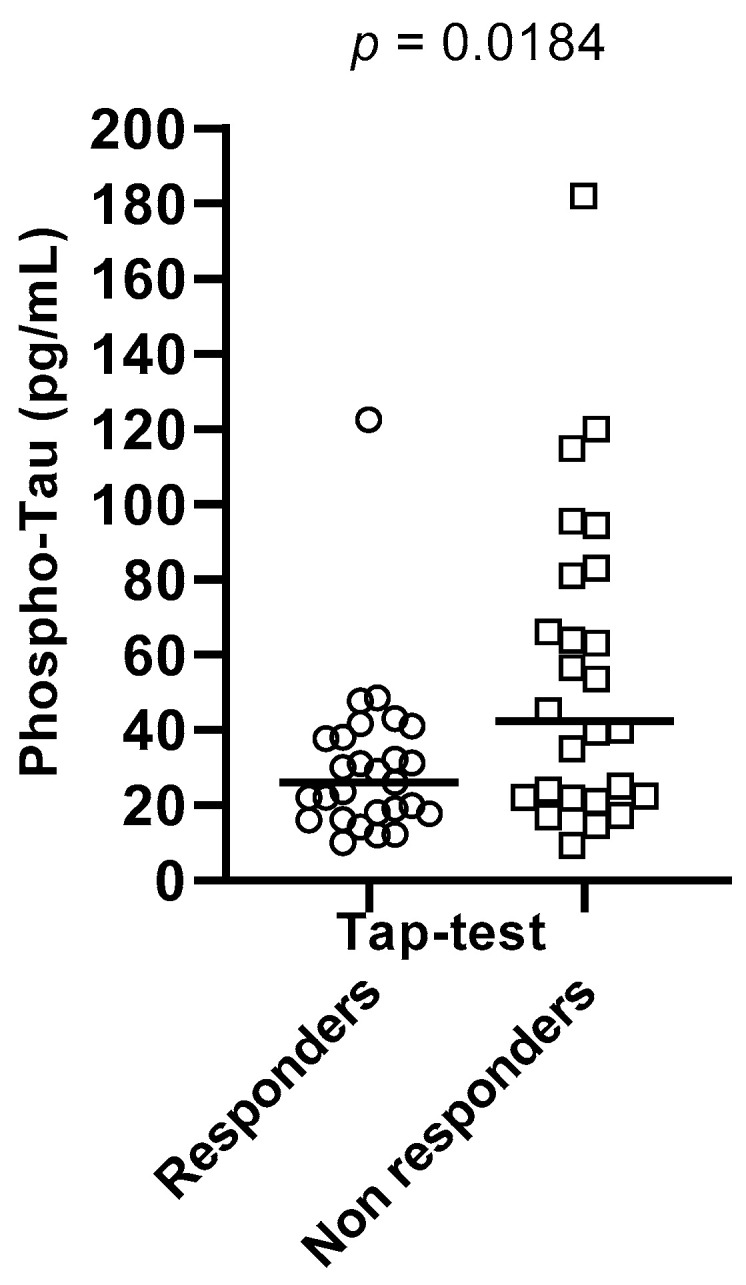
Phospho-Tau levels differed significantly between Tap-test responders (depicted in circles) and non-responders (depicted in squares) (*p* = 0.0184). The median values and the range of phospho-Tau values in the two groups are presented in this graph.

Aβ42/Aβ40 ratios were found to be significantly different between the two sub-groups (*p* = 0.0184), with Tap test responders having a higher value of ratio than non-responders. Total Tau and phospho-Tau CSF levels also differed significantly between these two groups (*p* = 0.0409 and *p* = 0.0184, respectively), with Tap-test responders having lower levels than non-responders. These results are depicted in Table 4 and Figure 1, Figure 2 and Figure 3.

The other two variants, namely Aβ42 and phospho-Tau/Aβ42, have not proved to differ significantly between Tap-test responders and non-responders.

**Figure 4:**
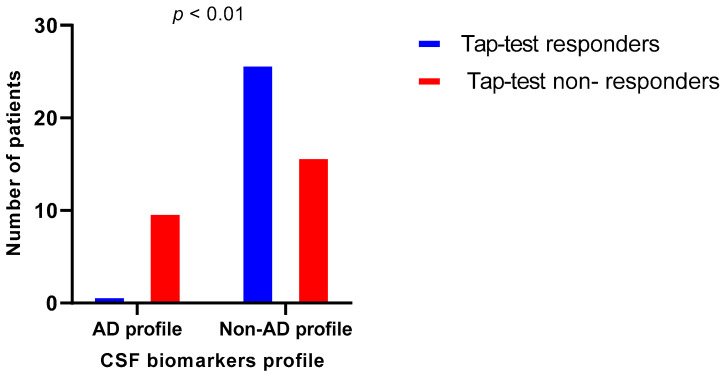
Number of patients with and without Tap-test response according to their CSF biomarker profile (AD or non-AD).

Then, in order to classify patients into two distinct biomarker profiles, CSF biomarkers were transformed into binary variables (normal or abnormal) based on cut-off values of the Unit of Neurochemistry and Biomarkers (Aβ42 < 480 pg/mL; total Tau > 400 pg/mL and phospho-Tau > 60 pg/mL), as previously described [31]. Patients with abnormal Aβ42, and/or Aβ42/Aβ40, total Tau and phospho-Tau were considered to have concomitant underlying AD pathology according to the BIOMARKAPD/ABSI criteria, and were classified in the group of patients with an AD profile [32,33]. All the rest were classified as having a non-AD profile. The demographic and clinical characteristics of these two groups are presented in Table 5. There is no significant difference between these two groups regarding gender, age, disease duration and pre-Tap-test clinical and neuropsychological status. Patients with an AD profile of CSF biomarkers have proved less likely to have a positive response to the Tap-test than patients with a non-AD profile (χ^2^ = 9.729, *p* < 0.01), as depicted in Figure 4.

## 4. Discussion

In the present study, we aimed to quantify established CSF biomarkers for AD indicating underlying AD pathology in patients with iNPH, and to explore their potential role of favorable prognosis with regard to shunting.

According to our results, no significant correlation was drawn between Aβ42 and Tap-test responsiveness. Findings on Aβ42 and its correlation with iNPH prognosis are controversial, with Kang et al. (2014) suggesting that lower Aβ42 levels are associated with worse cognitive outcomes, although they did not differ significantly between Tap-test responders and non-responders. However, Santangelo et al. (2017) found that there was no statistically significant difference in cognitive or gait/balance performance during CSF removal in line with Aβ42 levels [34,35]. On the other hand, Aβ42/Aβ40 ratios were found to be higher in Tap-test responders, which is in accordance with the findings of Kanemoto et al. (2021), who suggested that low values of CSF Aβ42/40 ratio are correlated with a poorer Tap-test outcome, especially in the cognitive field [36].

Aβ is fundamentally associated with the amyloid hypothesis of AD. Our current hypothesis considers that neurodegeneration is caused by an imbalance between the production of Aβ and its clearance within the brain, resulting in accumulation, plaque formation, and finally, cognitive decline [37]. AD and iNPH share pathophysiological mechanisms, and in the latter, CSF concentrations of both Aβ and soluble amyloid precursor protein (sAPP) fragments (with a probable trophic role for neurons) have been found decreased. These findings do not support a specific amyloid cascade mechanism, and are thought to be the result of a downregulation of sAPP in periventricular areas of the brain, which may be the result of impaired amyloid metabolism or reduced extracellular fluid clearance towards CSF [38]. Aβ42, although a specific biomarker for AD, has also been found to be decreased in other neurodegenerative and non-neurodegenerative diseases as well [39,40,41]. Thus, the Aβ42/Aβ40 ratio has been introduced, which is considered to correct for inter-individual variability in the overall Aβ production, possibly reflecting more accurately individual amyloid burden [42].

Regarding Tau proteins, we found that the concentrations of both total Tau and phospho-Tau were lower in patients with a positive response to the Tap-test. High concentrations of phospho-Tau have also been suggested as a possible marker of poor cognitive and overall outcomes after shunt surgery by Akiba et al. (2018) [43]. Total Tau is considered to be an unspecific marker of degeneration or axonal damage [37,44]. Phospho-Tau, on the other hand, is considered a biomarker with molecular specificity for AD, reflecting neurofibrillary tangle formation, and it is usually normal in other neurological diseases [39,40,45], including pure iNPH [46]. Increased CSF phospho-Tau concentration possibly reflects AD coexistence [47], while total Tau is a non-specific marker of neurodegeneration like many other molecules, such as 14-3-3 protein and NFs [48].

No significant correlation between the phospho-Tau/Aβ42 ratio and the Tap-test responder status has been found in the present study. Ray et al. (2011) and Patel et al. (2012) have correlated high values of this ratio with the possibility of developing a neurodegenerative dementia, mostly AD, and a worse outcome after shunt surgery [49,50]. On the contrary, Kang et al. (2014) suggested that higher values of phospho-Tau/Aβ42 were associated with a positive response to the Tap-test [34].

Regarding the full AD CSF profile, in the present study, it has been found that iNPH patients with an AD profile are less likely to have a positive response to the Tap-test than those with a non-AD profile. An earlier study by Golomb et al. (2000) had suggested that an AD profile in iNPH patients has little effect on the possibility of shunt responsiveness [13]. Another study by Lim et al. in 2014 concluded that iNPH patients with an AD profile were less likely to have a good response either to Tap-test or to shunt placement [51]. On the other hand, Müller-Schmitz et al. (2020) suggested that iNPH patients with an AD profile had better outcomes after CSF evacuation than those with a non-AD profile [52]. Nevertheless, our results seem to be in accordance with a recent meta-analysis, which concluded that elevated CSF phospho-Tau concentration is correlated with worse post-shunt outcomes [53].

Our study has certain limitations too. Patients included have no pathologic confirmation, an inherent disadvantage in the majority of relevant studies; there are also no established pathologic data for iNPH. Therefore, we used solid criteria based on the latest iNPH guidelines for the patients’ inclusion or exclusion in the study, as described above. Regarding concerns about the possible learning effect that could disturb the value of at least 10% simultaneous improvement in MMSE and FAB, Solana et al. (2010) have shown that iNPH patients do not have better performance in a setting of repeated cognitive tests when no other intervention is applied, unlike non-iNPH subjects [54]. Additionally, we have neither information about patients that were surgically treated nor post-shunt data. Another limitation is the relatively small number of patients, resulting from the fact that this is a one-center study. However, this might be a strength of the study too, as in this way, inter-rater variability regarding clinical and neuropsychological evaluation is avoided. Nevertheless, several statistically significant results have been drawn. Despite that, due to the small number of patients, differences regarding single biomarkers would not be retained if a post hoc analysis was applied. Further, multicenter well-conducted studies with larger patient cohorts and ideally post-surgical data are required in the future in order to validate or confute our results.

## 5. Conclusions

In conclusion, the results of the present study show that CSF biomarkers established for AD can be useful in iNPH patients too. Higher CSF levels of total Tau and phospho-Tau and lower values of Aβ42/Aβ40 ratio, as long as a total CSF biomarker profile indicative of AD pathology, seem to make a positive Tap-test response less likely. Future studies that will include post-surgical data are required in order to ascertain these results.

## Figures and Tables

**Table 1:** Demographic and clinical characteristics before LP of Tap-test responders and non-responders.

Variable	Tap-Test Responders N = 27	Tap-Test Non-Responders N = 26	*p*
Gender (F/M)	14/13	8/18	0.119 ^†^
Age	75 (69–77)	74.5 (70.75–77.25)	0.891 ^‡^
iNPH Grading scale	6 (5–7)	6 (4–7)	0.839 ^‡^
Disease duration (months)	24 (14–48)	24 (12–48)	0.903 ^‡^
MMSE before LP	23 (17–26)	23.5 (20.5–28.25)	0.062 ^‡^
FAB before LP	9 (8–13)	12 (9.75–15)	0.184 ^‡^
10 m timed walk test: steps before LP	27 (20–45)	19.5 (16–27.75)	0.04 ^‡,^*
10 m timed walk test: time before LP	17 (11–36)	10.75 (9–15)	0.02 ^‡,^*

N = number of subjects; LP = lumbar puncture. Demographic and clinical parameters are presented as median values (25th–75th percentile); ^†^ χ^2^ test; ^‡^ Mann–Whitney U test; statistically significant *p* values are marked with *.

**Table 2:** Neuropsychological and gait data of Tap-test responders before and 48 h after LP.

N = 27	Neuropsychological						Gait	
	MMSE	FAB	5WT Immediate	5WT Delayed	CLOX-1	CLOX-2	10 m Timed Walk Test: Steps	10 m Timed Walk Test: Time
Before LP	23 (17–26)	9 (8–13)	5 (5–5)	5 (4–5)	7 (4–11)	10 (8–13)	27 (20–45)	17 (11–36)
48 h after LP	25 (20–28)	12 (9–14)	5 (5–5)	5 (4–5)	8 (6–11)	12 (8–14)	22 (17–35)	14 (9–25)
Median % change	0.103	0.182	0.000	0.000	0.000	0.071	0.231	0.235
*p*	<0.001 ^†^	<0.001 ^†^	NS ^†^	NS ^†^	0.012 ^†^	0.011 ^†^	0.001 ^†^	<0.001 ^†^

N: total number of subjects, LP: lumbar puncture, 5WT: 5-word test, NS: non-significant, MMSE: Mini-Mental State Examination, FAB: Frontal Assessment Battery; Neuropsychological and gait data are presented as median values (25th–75th percentile), as they did not have normal distributions and homogenous variances. ^†^ Wilcoxon matched pairs test.

**Table 3:** Neuropsychological and gait data of Tap-test non-responders before and 48 h after LP.

N = 26	Neuropsychological						Gait	
	MMSE	FAB	5WT Immediate	5WT Delayed	CLOX-1	CLOX-2	10 m Timed Walk Test: Steps	10 m Timed Walk Test: Time
Before LP	23.5 (20.5–28.25)	12 (9.75–15)	5 (4.75–5)	4 (2–5)	9.5 (4.75–13)	12 (8–13.25)	19.5 (16–27.75)	10.75 (9–15)
48 h after LP	24.5 (19.5–29.25)	13 (10.75–15.5)	5 (4.75–5)	4.5 (1.75–5)	10 (4–12)	11 (7.5–13)	19 (15.75–29)	11 (8.38–18)
Median % change	0.017	0.000	0.000	0.000	0.000	0.000	0.024	0.038
*p*	NS ^†^	0.029 ^†^	NS ^†^	NS ^†^	NS ^†^	NS ^†^	NS ^†^	NS ^†^

N: total number of subjects, LP: lumbar puncture, 5WT: 5-word test, NS: non-significant, MMSE: Mini-Mental State Examination, FAB: Frontal Assessment Battery. Neuropsychological and gait data are presented as median values (25th–75th percentile), as they did not have normal distributions and homogenous variances. ^†^ Wilcoxon Matched Pairs Test.

**Table 4:** CSF biomarkers data of Tap-test responders and non-responders.

CSF Biomarker	Tap-Test Responders Ν = 27	Tap-Test Non-Responders Ν = 26	*p*
total Tau	194 (157–272.3)	272.2 (151.2–548.3)	0.0409 ^†^*
phospho-Tau	26 (17.7–38)	42.5 (21.97–81.5)	0.0184 ^†^*
Aβ42	389 (304–609.5)	406.2 (317.1–660.8)	0.5871 ^†^
Aβ42/Aβ40 ratio	0.16 (0.1–0.2)	0.11 (0.06–0.163)	0.0184 ^†^*
phospho-Tau/Aβ42 ratio	0.049 (0.037–0.106)	0.135 (0.036–0.211)	0.2116 ^†^

N: number of patients. CSF biomarker data are presented as median values (25th–75th percentile). ^†^ Mann–Whitney U test; statistically significant *p* values are marked with *.

**Table 5:** Demographic and clinical characteristics before LP of patients with an AD CSF biomarker profile and non-AD profile.

Variable	iNPH Patients with AD Profile N = 11	iNPH Patients with Non-AD Profile N = 42	*p*
Gender (F/M)	5/6	17/25	0.765 ^†^
Age	76 (66–82)	74.5 (70–77)	0.668 ^‡^
iNPH Grading scale	6 (4–7)	6 (5–7)	0.984 ^‡^
Disease duration (months)	24 (15–74.5)	24 (13–48)	0.960 ^‡^
MMSE before LP	23 (11–29)	24 (18.75–27)	0.252 ^‡^
FAB before LP	11 (5–14)	11.5 (9–13)	0.640 ^‡^
10 m timed walk test: steps before LP	22 (15–31)	25 (18.5–33)	0.199 ^‡^
10 m timed walk test: time before LP	14 (8–15)	14.25 (10–24)	0.640 ^‡^

N = number of subjects; LP = lumbar puncture. Demographic parameters are presented as median values (25th–75th percentile); ^†^ χ^2^ test; ^‡^ Mann–Whitney U test.

## Data Availability

The data presented in this study are available upon reasonable request from the corresponding author. The data are not publicly available due to privacy restrictions.
